# Diseases of the Nervous System

**Published:** 1892-10

**Authors:** 


DISEASES OF THE NERVOUS SYSTEM.—By Jerome K. Bauduy, M. D., LL. D., Professor of Diseases of the Mind and Nervous System, and Medical Jurisprudence, Missouri Medical College, St. Louis; late Physician in Chief to St. Vincent's Institution for the Insane; corresponding member of the New York Society of Neurology and Electrology, etc. Published by J. B. Lippincott Co., Philadelphia.

PROFESSOR BAUDUY has completed quite a number of quotations and formulated them into a book, adding a few italics as the author's own, and in doing so, we fear, has not done himself justice, for we think he could have done better had he given his knowledge to the world, as many others have done and with credit to themselves in the form of clinical lectures, where he could have better displayed his knowledge, unaided by quotations.

This review would not be severe, but only attempts a suggestion to those ambitious authors: that rather one original suggestion, or discovered truth, than all the world of known facts or hypotheses, enclosed between the backs of a volume of many pages, for it is well known that a book does not make fame, or add to the knowledge of the reading world unless it contains new facts or suggestions. H. H.

